# Vertebral Aneurysmal Bone Cyst Mimicking Osteosarcoma: Case Report and Review of the Literature

**DOI:** 10.7759/cureus.35033

**Published:** 2023-02-15

**Authors:** Elizabeth Lechtholz-Zey, Nawar Y Matti, Vance L Fredrickson, Ian Buchanan, Daniel A Donoho

**Affiliations:** 1 Orthopaedic Surgery, Keck School of Medicine of University of South California, Los Angeles, USA; 2 Pathology, Southwest Healthcare System, Los Angeles, USA; 3 Neurosurgery, Billings Clinic, Billings, USA; 4 Neurosurgery, Mayo Clinic, Jacksonville, USA; 5 Neurological Surgery, Children's National Hospital, Washington, DC, USA

**Keywords:** benign bone lesion, spinal instrumentation, pediatric, thoracic spine, surgical oncology, aneurysmal bone cyst

## Abstract

Aneurysmal bone cysts are benign osseous lesions containing blood-filled cavities separated by walls of connective tissue. They can be difficult to identify clinically due to similarities in presentation, imaging, and histology with other pathologies. Specifically, it is important to distinguish these benign lesions from malignant processes, as both surgical and medical management differ. We present the case of a 21-year-old patient who presented with impaired motor and sensory function in his lower extremities. Radiologic findings were concerning for an invasive neoplasm, and the intraoperative frozen section supported this conclusion. However, an additional histological investigation was confirmatory for a diagnosis of an aneurysmal bone cyst. The patient underwent corpectomy, laminectomy, and a posterior spinal fusion, and regained motor and sensory function shortly thereafter. This report details the importance of considering aneurysmal bone cysts in the differential of infiltrative bone lesions, despite their benign nature, as medical and surgical management can vary greatly.

## Introduction

In 1942, physicians Henry Lewis Jaffe and Louis Lichtenstein first described two cases of an odd blood-filled bone lesion in patients presenting with advanced neurologic deficits, coining the term aneurysmal bone cyst (ABC) [1]. However, scientific inquiry has revealed that these lesions are neither bone cysts nor aneurysms [2]. ABCs are benign bone lesions with blood-filled cavities separated by walls of connective tissue. They are most common in the pediatric population and can occur in any bone, but are more typically found in the spine, knee, or pelvis [2-4]. The presence of a cystic lesion with fluid-fluid levels on computed tomography or magnetic resonance imaging should raise suspicion of an aneurysmal bone cyst [5]. ABCs occur in the population at a rate of 0.15 cases per million people per year, [6] and vertebral ABCs are amongst the more frequently diagnosed subtypes, comprising between 3-20% of all subtypes [7]. When located in the spinal column, ABCs tend to cause a mixture of somatosensory and motor symptoms in a dermatomal distribution and are typically limited to involvement in just one vertebra, which may be helpful in distinguishing these lesions from other cancerous processes, including osteosarcomas and hemangiomas [8]. The natural history of ABCs, including their propensity to rehemorrhage and expand in size, raises the specter of acute neurologic harm from these lesions. ABCs near the spinal cord can be more technically difficult than those of the extremities due to the inherent anatomic complexities and propensity for damage to nearby structures [9]. In addition to surgical resection, radiotherapy and curettage are amongst treatment options for these bone lesions, as well as some novel therapies such as denosumab, bisphosphonates, doxycycline, and sclerotherapy [5,9-10]. Even after surgical treatment, ABCs have the potential for recurrence, although this rate is variable amongst the literature [6].

## Case presentation

**Figure 1:**
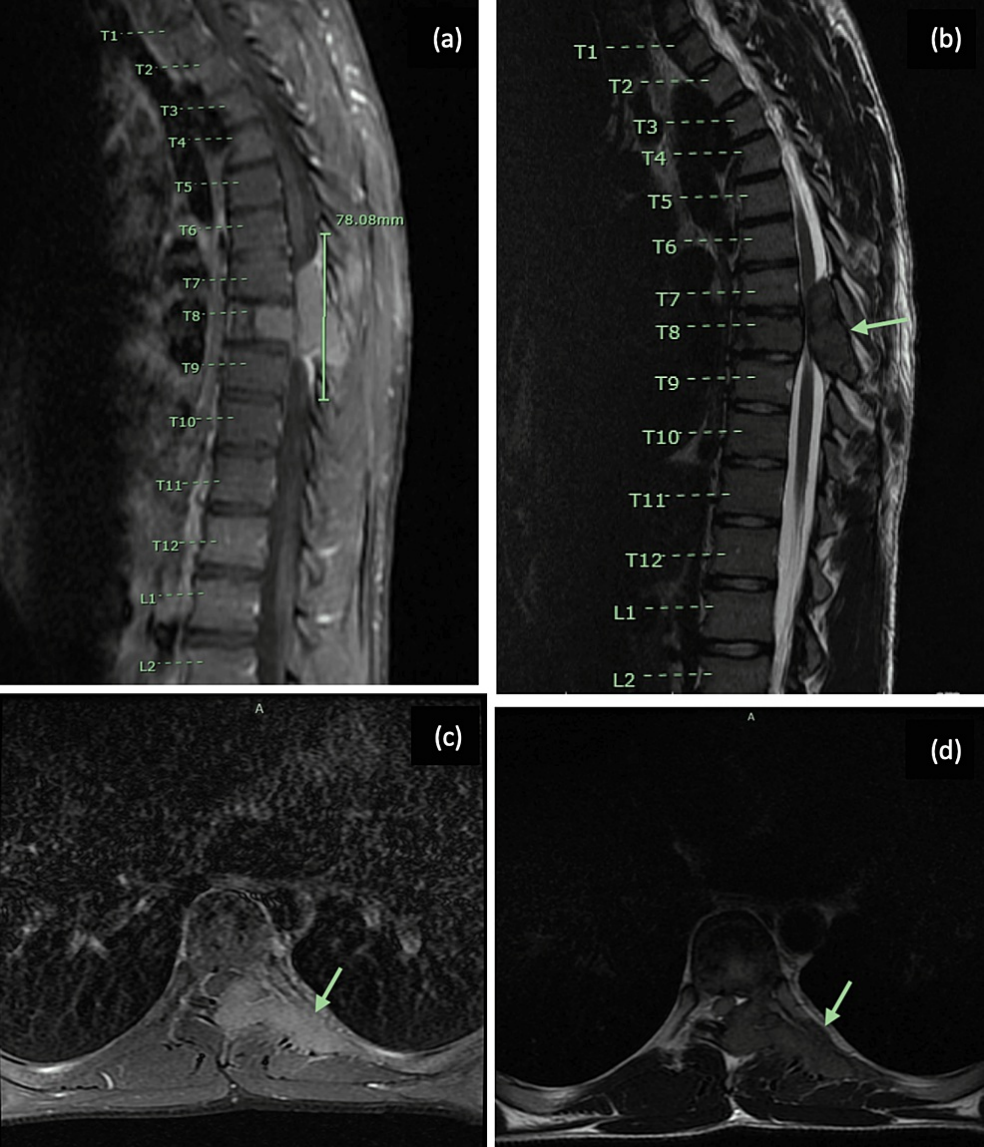
Pre-operative MRI showing a diffuse, marrow-replacing lesion at T8 with resulting effacement into the T7-T8 CSF spaces and severe spinal cord compression. (a) Sagittal T1-weighted fat-saturated post-contrast image. (b) Sagittal T2-weighted image. (c) Axial T1-weighted fat-saturated post-contrast image. (d) Axial T2-weighted image.

A 21-year-old male presented to the emergency department with severe back pain and the inability to walk for five days. He had not had prior trauma and did not have a significant personal or family medical history. He had a spontaneous pneumothorax 18 months prior which resolved without intervention. A chest computed tomography (CT) image was obtained during that hospitalization with no evidence of any extra-pulmonary processes. On physical exam during the present admission, the patient demonstrated 5/5 motor strength in his upper extremities bilaterally, but he had 1/5 strength on bilateral lower extremity dorsiflexion and testing of the extensor hallucis longus, as well as 4/5 strength on plantarflexion, hip flexion, and knee extension bilaterally. Additionally, he exhibited 4-5 beats of ankle clonus bilaterally and decreased sensation to light touch in the T9-T10 dermatomes bilaterally. Magnetic resonance imaging (MRI) with contrast using both T1- and T2-weighted sequences revealed a diffuse marrow-replacing process with complete infiltration throughout the T8 vertebra, as well as resulting effacement of the T7-T8 CSF spaces and severe spinal cord compression (Figure 1). The patient had no evidence of other craniospinal abnormalities on MRI. 

**Figure 2:**
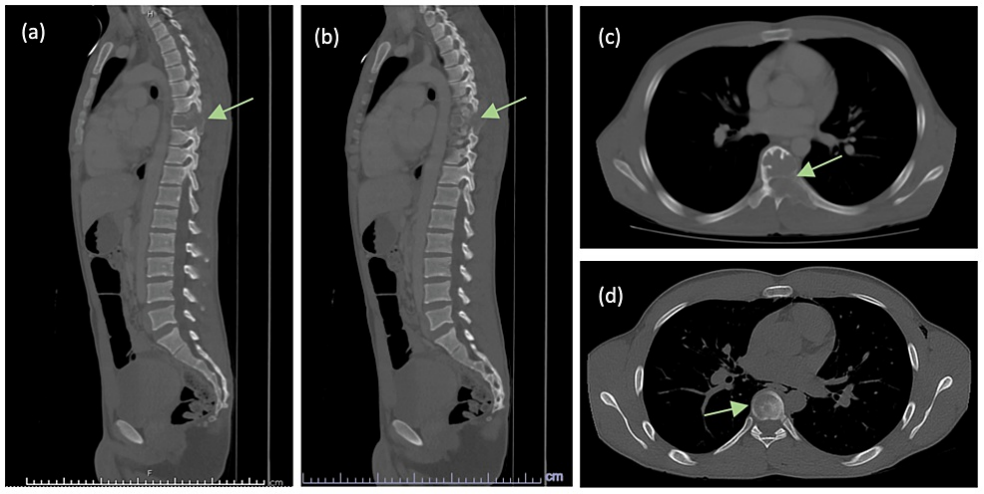
Computed tomography images demonstrating an expansile soft tissue mass in the eighth thoracic vertebra. (a) Sagittal view of soft tissue mass involvement of the T8 vertebral body, left pedicle, and spinous process. (b) Sagittal view demonstrating soft tissue mass extension into surrounding structures. (c) Axial view demonstrating involvement in the T8 left pedicle, left transverse process, and left lamina. (d) Axial view highlighting involvement of the T8 vertebral body.

**Figure 3:**
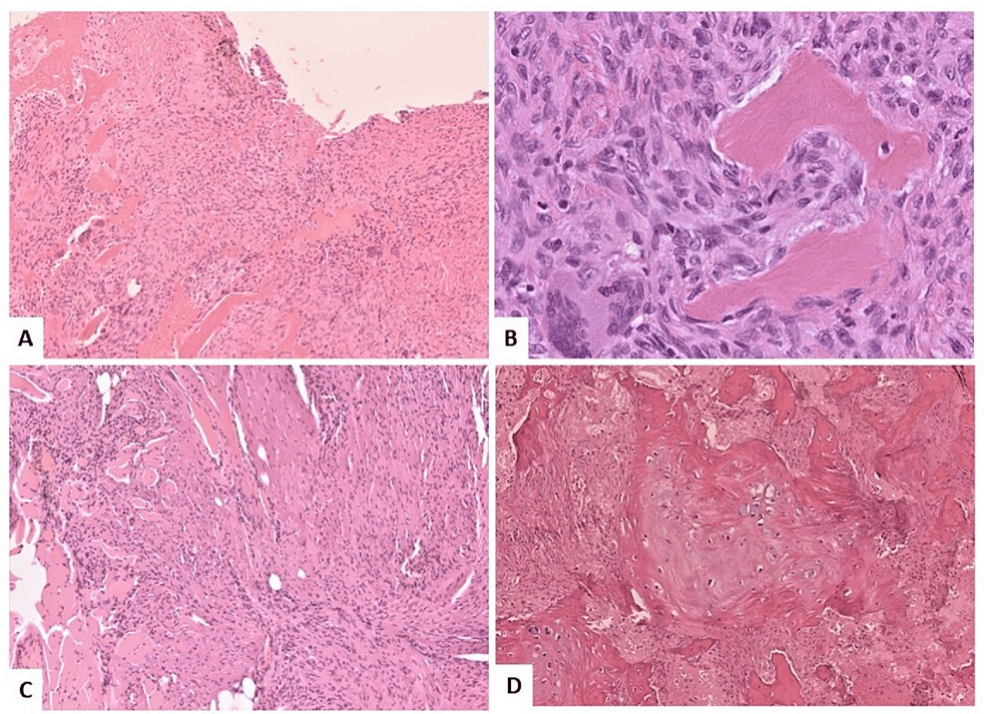
H&E stained slides of post-operative pathology specimen showing a thoracic solid aneurysmal bone cyst. (A) Low power 4x magnification showing cyst wall with spindle cell proliferation (fibroblasts), multiple giant cells, reactive bone formation and hemosiderin deposition. No blood lakes are seen consistent with the solid pattern. (B) High power 40x magnification showing benign spindle cell features with no cytologic atypia or atypical mitoses present. (C) 4x magnification showing that lesion is locally involving the skeletal muscle causing destruction and atrophy. (D) 4x magnification showing partially calcified immature woven bone trabeculae.

A subsequent CT was compared to the previous scan performed a year and a half prior to the case in question and showed a remarkable difference in the findings related to the thoracic spine, indicating a rapidly growing and expansile lesion at T8, felt most likely to be an osteosarcoma (Figure 2). Due to the patient’s Bilsky grade 3 lesion, the neurosurgical service was consulted and surgery was recommended. We performed a T8 corpectomy with costotransversectomy, T7-T9 laminectomy, and T6-T10 posterior spinal fusion. We planned an extracapsular approach to allow complete resection of the mass. During surgery, a large paraspinal mass was easily identified and observed to be highly vascular. The total operative time was eight hours and five minutes, and surgical blood loss was 2 liters, which was unexpected based upon the initial frozen section and radiographic features of the tumor. The anaesthetic team was well prepared with excellent intravenous access and transfused a total of seven units of packed red blood cells, three units of platelets, and four units of fresh frozen plasma. Tissue sections were retrieved intraoperatively, and the initial report of the frozen section identified the left paraspinal mass as a spindle cell carcinoma. However, the final pathologic diagnosis identified this mass as a solid aneurysmal bone cyst with the thoracic tumor having additional skeletal muscle involvement (Figure 3). 

**Figure 4:**
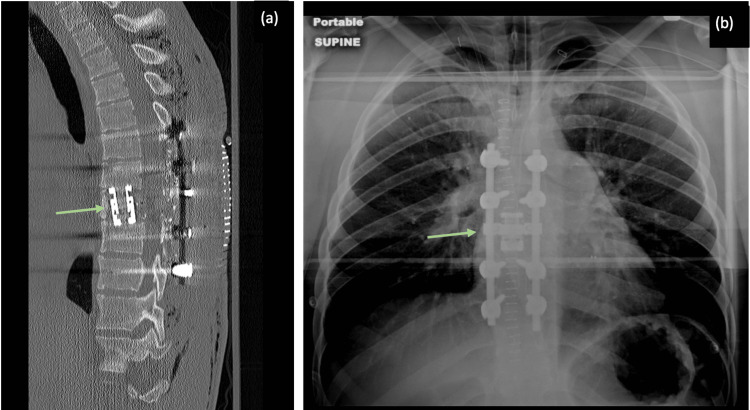
Post-operative images confirming appropriate hardware placement. (a) CT image demonstrating appropriate corpectomy cage at T8. (b) Chest X-ray demonstrating posterior hardware fixation spanning T6-T10 with corpectomy cage at T8 and laminectomies spanning T6-T9.

**Figure 5:**
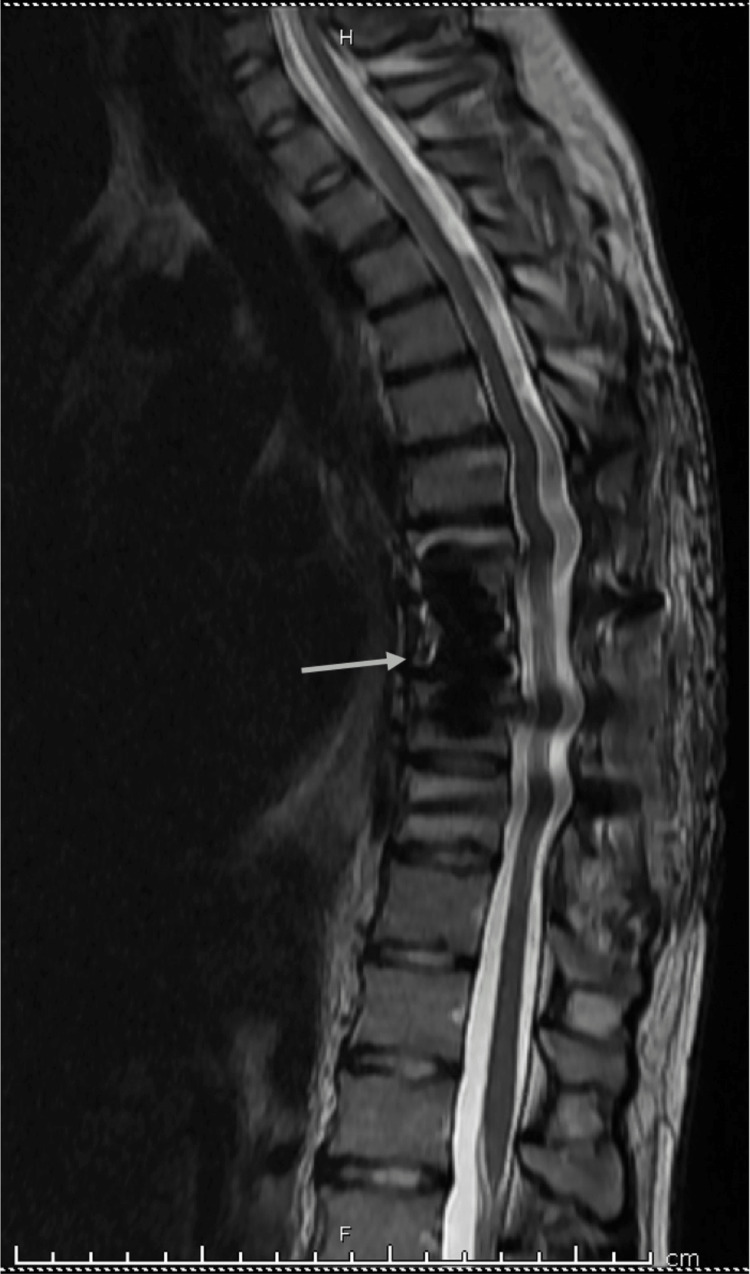
Six-month post-operative T2-weighted MRI demonstrating appropriate hardware fixation with no evidence of recurrence of the vertebral body mass or spinal cord compression.

A CT of the thoracic spine obtained immediately after the operation demonstrated appropriate placement of the corpectomy cage at the T8 level with posterior hardware fixation spanning from T6-T10 (Figure 4). There was a noted improvement in spinal canal patency as compared to the pre-operative MRI. Physical exam findings gradually improved on each subsequent post-operative day, and the patient was determined to not be a candidate for inpatient rehabilitation given his high level of functioning. The patient regained full strength and sensation in bilateral lower extremities within several days of surgery and was discharged on post-operative Day 6 with no post-operative complications. Continuous imaging over the next several months showed proper hardware placement with no evidence of recurrence, as well as a patent spinal canal with no areas of abnormal enhancement. At six months after surgery, he is symptom-free without any residual neurological deficits and has no indication of a residual or recurring aneurysmal bone cyst (Figure 5).

## Discussion

In the case of our 21-year-old patient with a Bilsky grade 3 aneurysmal bone cyst of the thoracic spine, complete surgical resection with stabilization via a corpectomy cage proved to be an effective management strategy with excellent clinical and patient-reported outcomes. Extensive diagnostic workup ultimately distinguished this patient’s lesion from a malignant process, but aneurysmal bone cysts are disproportionately underrepresented in radiographic differential diagnoses [11]. While aneurysmal bone cysts can be locally aggressive, they are typically constrained to their bony surroundings. In this case, the invasive nature of the lesion led to the initial diagnosis of sarcoma and subsequent surgical planning for cancer surgery. Osteosarcoma was especially high on the differential because of the invasion of the mass into the paraspinal skeletal muscle, as well as the age of the patient. Additionally, the patient’s imaging studies showed only modest enhancement after administration of contrast, which when coupled with the lack of fluid-fluid levels, further increased the likelihood of an osteosarcoma. Specifically, telangiectatic osteosarcomas may be confused for aneurysmal bone cysts since they tend to occur in similar locations and resemble each other radiographically, grossly, and even on histopathology [12]. Ultimately, the pathology and gross visualization of the lesion confirmed a benign, solid aneurysmal bone cyst of the thoracic spine.

The management of ABCs is highly dependent on patient characteristics, including age, degree of neurological involvement, surgical accessibility, and ability to minimize surgical blood loss, as well as consideration for vertebral instability post-operatively [5,9-10]. When complete resection is possible, surgical management is the preferred option, as these tumors are known to recur from incomplete resections or curettage alone. Embolization is a commonly employed method of reducing vascularity and can reduce intraoperative blood loss if used as a preoperative adjunct in carefully selected patients. Monotherapy with embolization has had varying success in the complete resolution of ABCs, but this application has typically been in the context of flat bones [9]. For patients who are unable to tolerate surgery or who have recurrent lesions, repeat percutaneous embolization may be the most appropriate treatment modality. Surgical management consists of curettage or complete excision. Curettage carries a risk of overall local recurrence as high as 50%, though this rate is variable within the literature, and is not considered to be an optimal treatment option [5]. An effective strategy may be to perform preoperative embolization prior to wide local resection of the ABC in order to minimize intraoperative blood loss [4]. Overall, complete excision with subsequent surgical stabilization carries the best prognosis for patients with neurological deficits [9,13]. 

Several recent investigations [14-16] suggest promising evidence for conservative treatment of both primary and recurrent ABCs without neurologic deficits using modalities such as denosumab, bisphosphonates, percutaneous doxycycline, and sclerotherapy [10]. Though case reports [17-19] indicate that these may be viable options, larger sample sizes with frequent and longer follow-ups are required before official recommendations can be made. Patients who have been successfully treated with these more conservative options did not demonstrate significant neurological deficits, which would necessitate surgical management for decompression [20]. 

An ABC masquerading as a sarcoma placed a significant amount of importance on the pathological interpretation of the tissue sample, yet particular consideration to the safety of biopsy must be given in the context of a highly vascularized lesion with the potential for significant blood loss. Given the difficulty in establishing a diagnosis of an aneurysmal bone cyst based on radiographic evaluation alone, there is general agreement that a biopsy should be performed for a definitive diagnosis [3,9-10]. The need for biopsy may need to be reconsidered in those patients who face a higher risk of neurologic harm from hemorrhagic expansion. The initial diagnosis of a spindle cell neoplasm would have required a vastly different clinical and surgical approach, including oncological surgical planning and a more extensive pre-operative evaluation. ABC can be managed via nonsurgical intervention unless spinal instability, intractable pain, or neurologic deficits are present, as in this case. Here, gross total resection of the ABC allows for the best possible neurologic outcome. 

## Conclusions

Aneurysmal bone cysts (ABC) of the vertebrae are a rare but important diagnostic consideration in the workup of a young patient with a vertebral column mass. Unlike systemic malignancies or sarcoma, aneurysmal bone cysts of the spine are a proliferative, hemorrhagic bone disease that can be cured with surgical resection without requiring oncologic margins. Upon initial radiographic analysis, ABC can disguise itself as an osteosarcoma or hemangioma, as in this case. Careful histopathological evaluation is required to properly distinguish an ABC from a cancerous process, as the surgical and perioperative management of an ABC is considerably different than that of a malignant neoplasm. ABC should be considered during the evaluation of paraspinal masses even when classical radiographic findings are not present.
